# Transcriptomics of Upper Gastrointestinal Fluids Can Diagnose Pancreatic Ductal Adenocarcinoma

**DOI:** 10.1016/j.jcmgh.2026.101776

**Published:** 2026-03-26

**Authors:** Tal Barkai, Oran Yakubovsky, Keren Bahar Halpern, Ido Nachmany, Shalev Itzkovitz, Bilomics Research Group, Alon Israeli, Michal Fine, Noa Oren, Paz Kelmer, Niv Pencovich, Ron Pery

**Affiliations:** Department of Molecular Cell Biology, Weizmann Institute of Science, Rehovot, Israel; Sheba Medical Center, Ramat-Gan, Israel; Department of Molecular Cell Biology, Weizmann Institute of Science, Rehovot, Israel; Gray Faculty of Medical and Health Sciences, Tel-Aviv University, Tel-Aviv, Israel; Department of Surgery and Transplantation, Sheba Medical Center, Ramat-Gan, Israel; Department of Molecular Cell Biology, Weizmann Institute of Science, Rehovot, Israel; Gray Faculty of Medical and Health Sciences, Tel-Aviv University, Tel-Aviv, Israel; Department of Surgery and Transplantation, Sheba Medical Center, Ramat-Gan, Israel; Department of Molecular Cell Biology, Weizmann Institute of Science, Rehovot, Israel

Noninvasive diagnostics of cancer can enable early detection thus improving treatment success rates. This goal is particularly critical for rapidly progressing tumors such as pancreatic ductal adenocarcinoma (PDAC).^1^ Genomics, epigenomics, and transcriptomics of blood-based liquid biopsies demonstrated statistical power in PDAC diagnosis, yet sensitivity of these methods was challenging at early stages.^2^ The upper gastrointestinal (GI) tract, including the esophagus, stomach, and duodenum, sheds massive amounts of cells every day into the upper GI tract lumen. Given that cancer induces systemic alterations in patients, such as cachexia, hormonal shifts, and even modifications in gene expression in central organs,^3^ we explored whether the patterns of cell shedding in upper GI tract fluids could be statistically significant for diagnosing PDAC.

We analyzed a cohort of 24 patients with PDAC and 30 controls. The control patients’ fluid transcriptomics were obtained from Barkai et al.^4^ and the fluids from patients with PDAC were generated for this study. The control and patients’ samples were collected, processed, and sequenced under identical conditions. Our cohort consisted of 29 males (54%) and 25 females (46%) with a mean age of 61.4 years. Most of the patients with PDAC (83%) had resectable disease, indicating early-stage PDAC. For all patients, we aspirated fluids from nasogastric tubes, routinely placed during surgical procedures (Figure 1*A*). Fluids were enriched for biliary content, indicated by their green hue, to capture duodenal shed cells in addition to shedding originating from the stomach and esophagus. We used mcSCRBseq, a sensitive Unique Molecular Identifier (UMI) method for sequencing the host mRNA molecules. Our analyzed cohort included samples with more than 3000 genes (median of 184,053 UMIs per sample). Clustering of the upper GI fluid samples yielded 2 clusters, 1 significantly enriched in control patients (Figure 1*B*, *blue dendrogram*; hypergeometric *P* = 9.77e−5) and the second significantly enriched in patients with PDAC (Figure 1*B*, *red dendrogram*; hypergeometric *P* = 9.77e−5). The control-enriched cluster showed elevated levels of genes highly expressed in the esophagus, such as *SPRR3* and *KRT13* (*green branches*), whereas the PDAC-enriched cluster showed elevated levels of stomach pit cell genes (*GKN1* and *GKN2*, *pink branches*)^5^ (Figure 1*B*). We found that 4 of 5 of the patients with PDAC that clustered with the control cluster (*blue*) had no positive lymph node in their pathological specimen (Supplementary Table 1). To assess the potential changes in relative representations of different upper GI tract cell types in the shed cell fractions, we performed computational deconvolution of the fluid transcriptomics using CellAnneal^6^ based on signatures of human organs of the upper GI tract.^5^ This method models each bulk fluid sample as a mixture of cell types and estimates the proportion of each cell type by comparing the sample’s gene expression profile to cell type-specific reference signatures. Using this approach, we identified significant elevation in the estimated proportion of mature pit cells in patients with PDAC compared with controls (Figure 1*C*). We trained a linear classifier based on iteratively subdividing the data into 60% training and 40% test sets. In each iteration, we identified genes that were elevated and genes that were decreased in PDAC sample fluids, and computed a ‘PDAC score’ as the fractional sum of the genes elevated in the PDAC samples (Methods). Our classifier achieved an average area under the receiver operating characteristic curve of 0.74 (Figure 1*D*). We extracted a classification signature comprising genes that appeared in more than 50% of the training test set iterations. This consensus set included 156 genes with increased expression and 154 genes with decreased expression in the upper GI tract fluids of patients with PDAC compared with controls (Supplementary Table 1; Figure 1*E*). The median PDAC-to-control expression ratio was 1.5 for the 156 PDAC fluid up-regulated genes, and 0.68 for the 154 PDAC fluid down-regulated genes. Consistent with our computational deconvolution results, the classification signature genes with increased expression in PDAC fluids included classic gastric pit cell markers, such as *MUC5AC*, *GKN1*, *GKN2*, *TFF1*, and *TFF2* (Figure 1*E*). Beyond classification of PDAC vs controls, our analysis also exposed a marginally significant elevation in the PDAC score in patients with lymph node metastasis in patients with PDAC (*P* = .07) (Figure 1*F*).

**Figure 1:**
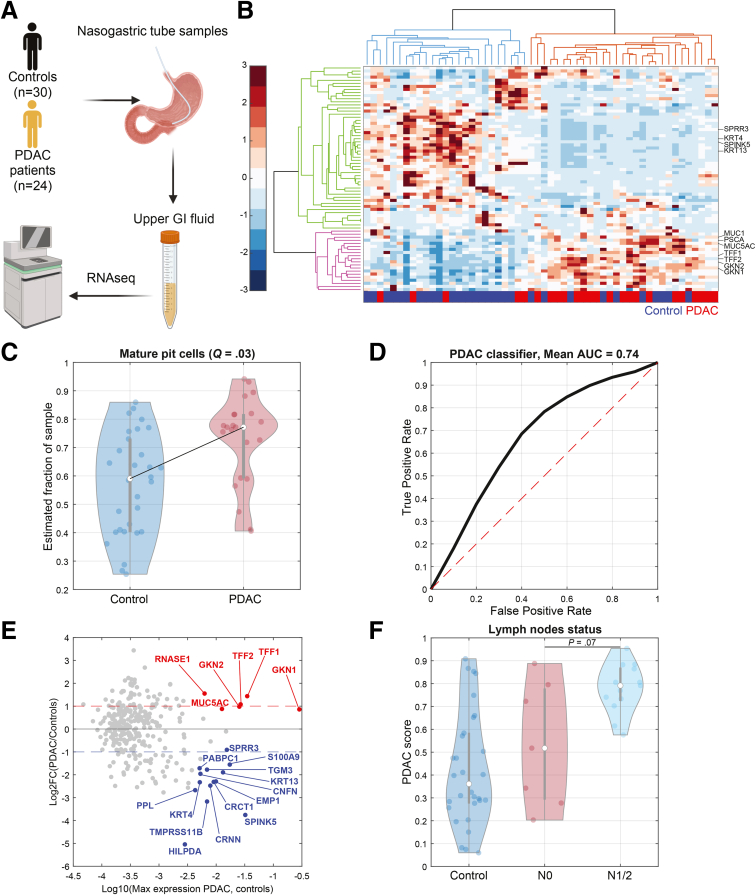
**Upper GI fluids transcriptomics detects PDAC.** (*A*) Outline of the study. Nasogastric tube (NGT) fluids were sampled from NGTs of patients with PDAC and controls, followed by RNA extraction and mRNA sequencing. Created in BioRender. (*B*) Clustergram of Z transformed sum normalized gene expression, showing clusters of patients with PDAC (*red*) and controls (*blue*), and their associated gene clusters (*pink and green*, respectively). Only genes with median expression of 1e−3 were included in the analysis. (*C*) Violin plot of computational deconvolution showing elevation in the estimated fractions of Mature pit cells in upper GI fluids from patients with PDAC. (*D*) Mean receiver operating characteristic (ROC) curve of 1000 data splits (see text, methods), with mean AUC of 0.74. (*E*) MA plot of the classifier markers. *Dashed red* and *blue lines* denote ratios of 2 and 0.5, respectively. Selected markers are shown in *red* for PDAC up-regulated markers, and in *blue* for PDAC down-regulated markers. Pseudonumber of 1e−6 was used for ratio calculation. (*F*) Violin plot of PDAC score as function of lymph node involvement. Wilcoxon rank sum test was performed between patients with PDAC without lymph node involvement (N0) to those with lymph node involvement (N1/N2); *P* = .07.

Our study demonstrates that shed cells from the upper GI tract, as sampled by nasogastric tubes, carry statistically powerful information on the appearance of PDAC. Although some of the elevated genes in the upper GI fluids from patients with PDAC overlap the metaplastic signatures of PDAC cells,^7^ the high expression levels and the overlap with pit cell genes render it unlikely that they originate from cells shed directly from the pancreas. Rather, the distinct transcriptomic signatures likely mirror variations in cell shedding patterns in the organs of the upper GI tract. PDAC is notorious for its systemic effects, including loss of appetite and neuropathic pain, elicited by changes in hormonal levels^8^ and interactions with neurons.^9^ Differential stimulation of the esophageal and gastric mucosa or muscle atrophy and decreased contractility associated with cancer^8^ could change the patterns of cell shedding, yielding the increased shedding of pit cells. Shed cell transcriptomics in fecal matter has been shown to be a powerful classifier of lower GI tract pathologies such as inflammatory bowel disease.^10^ Similarly, our study demonstrates that upper GI fluids carry statistical power for PDAC diagnosis. Because Whipple surgeries are performed at a relatively early stage of the disease, when the tumor is still operable, our approach could enable minimally invasive early PDAC diagnosis. The ease of obtaining these fluids, even without the involvement of a gastroenterologist, renders them particularly attractive for screening and diagnostic procedures of high-risk populations (BRCA carriers, family history, etc). Future studies will extend this cohort and explore the specificity of the shed cell transcriptomics signatures to distinguish upper GI tract tumor subtypes and the complementary use and comparison of these measurements with blood-based liquid biopsies.

## Supplementary Material

Supplementary Table 1

Extended PDF
