# Supplementary material for: Transcriptomics of Upper Gastrointestinal Fluids Can Diagnose Pancreatic Ductal Adenocarcinoma
Source: Cell Mol Gastroenterol Hepatol. 2026 Mar 26;20(7):101776. doi: 10.1016/j.jcmgh.2026.101776 (PMC13147430; doi:10.1016/j.jcmgh.2026.101776)
Supplement: Extended PDF [file mmc2.docx]

**Methods**

*Upper GI fluids collection*

Patients scheduled for a medical procedure were recruited from the General Surgery Department at Sheba Medical Center (Helsinki approval #SMC-8665-21). The study included patients undergoing abdominal surgery, for whom a NGT was routinely placed, independent of study participation, as well as patients with a pre-existing indwelling NGT. Clinical and demographic data was obtained from patients’ electronic health records. Samples from patients with evidence of pancreatic malignancy, as well as controls, were included in the current study. Control patients did not have upper GI malignancies, but rather various conditions ranging from healthy kidney donors, through benign adhesions, hernias, hepatobiliary pathologies, and colorectal cancer without upper GI metastases. Upper GI tract fluids were collected directly from the NGTs upon appearance of fluids with green hue, evidence for biliary secretion that should enrich for duodenal shed cells. All samples were processed within 20 minutes of collection. Samples were delivered to the lab in 15mL tubes on ice. At the lab, samples were centrifuged at 580g for 10 minutes at 4°C, the supernatant was removed, and the pellet was frozen immediately on dry ice and kept in -80°C until further processing.

*RNA extraction*

Tri-reagent was added at a ratio of 1:3, and samples were thawed on ice and thoroughly mixed every few minutes. Samples with a significant presence of solid content underwent additional centrifugation at this stage at 500g for 5 minutes at 4°C. Ethanol was added at a ratio of 1:1 to the supernatant, and extraction was continued according to the manufacturer’s instruction of Direct-zol micro prep kit (ZYMO research, R2062).

*Bulk RNA sequencing of the samples*

The mcSCRBseq protocol was used with minor modifications. Reverse transcription was applied on up to 500ng of RNA, with a final volume of 20μL (1×Maxima hour Buffer, 1 mM dNTPs, 2 μM TSO* E5V6NEXT, 7.5% PEG8000, 20U Maxima H enzyme, 2 μL barcoded RT primer). Subsequent steps were applied as mentioned in the protocol. Library preparation was performed using Nextera XT kit (Illumina) on 1-2.5ng amplified cDNA. Library final concentration was 1.8-2nM, and sequencing was done using the Novaseq 6000 (Illumina) sequencing machine aiming at 40M reads per sample with the following settings: Read1-16bp, Index1-10bp, Index2-10bp, Read2-66bp, few samples were sequenced with Index1-8bp, Index2-8bp.

*Bioinformatics and computational analysis of bulk RNA sequencing data*

Illumina output sequencing raw files were converted to FASTQ files using the UTAP pipeline. To obtain the UMI counts table, UTAP for SCRB-seq was used with CUTADAPT, and FASTQ files were aligned to the human reference genome (GRCh38). Data filtration and statistical analysis were performed with MATLAB R2024a and Python 3.9 using Scanpy 1.9.3 packages. Only protein-coding genes were retained, and mitochondrial and ribosomal genes were removed. Biomart for reference genome GRch38 V91 was used for gene classification as protein-coding. NGT samples with more than 3,000 genes were included in the analysis. Gene expression for each sample was normalized by the sum of the UMIs of each sample.

Cellular decomposition using Cellanneal

Computational cellular decomposition of bulk RNA sequencing data was performed using Cellanneal^6^ using signature tables based on Busslinger et al.^5^ single cell atlas of the human upper GI tract, using default parameters. Statistical analysis performed only on samples with spearman correlation > 0.3. Wilcoxon rank-sum test was performed between PDAC patients and controls, and multiple hypothesis correction was performed using Benjamini-Hochberg method.

*PDAC score classifier*

PDAC score was calculated using the following formula:

$$PDAC score=\frac{\sum PDAC genes}{\sum PDAC genes+\sum Control genes}$$

To establish diagnosis of PDAC patients, a linear classifier was constructed based on 1000 iterations. Within each iteration the data was divided into training and test sets, consisting of 60% and 40% of the data, respectively. PDAC and control genes were extracted from the training set as genes with median expression above 1e-4 and ratio (mean PDAC/mean control) above or below 1.5, respectively. Pseudonumber of 1e-6 was added to both mean when the ratio was calculated. Aggregate ROC was summarized by computing the median true positive rate (TPR) across false positive rate (FPR) bins and smoothing the resulting curve. Table S1 presents 156 PDAC genes and 154 control genes that appeared in the classifier gene sets at least 50% of the iterations.
